# Blood pressure and heart rate responses to orthostatic challenge and Valsalva manoeuvre in mild cognitive impairment with Lewy bodies

**DOI:** 10.1002/gps.5709

**Published:** 2022-04-07

**Authors:** Calum A. Hamilton, James Frith, Paul C. Donaghy, Sally A. H. Barker, Rory Durcan, Sarah Lawley, Nicola Barnett, Michael Firbank, Gemma Roberts, John‐Paul Taylor, Louise M. Allan, John O’Brien, Alison J. Yarnall, Alan J. Thomas

**Affiliations:** ^1^ Translational and Clinical Research Institute, Biomedical Research Building, Campus for Ageing and Vitality Newcastle University Newcastle upon Tyne UK; ^2^ Population Health Sciences Institute Newcastle University Newcastle upon Tyne UK; ^3^ College of Medicine and Health, St Luke's Campus Exeter University Exeter UK; ^4^ Department of Psychiatry University of Cambridge, Level E4, Addenbrooke's Hospital Cambridge UK

**Keywords:** Alzheimer's disease, dementia with Lewy bodies, mild cognitive impairment, orthostatic hypotension

## Abstract

**Objectives:**

Orthostatic hypotension is a common feature of normal ageing, and age‐related neurodegenerative diseases, in particular the synucleinopathies including dementia with Lewy bodies. Orthostatic hypotension and other abnormal cardiovascular responses may be early markers of Lewy body disease. We aimed to assess whether abnormal blood pressure and heart rate responses to orthostatic challenge and Valsalva manoeuvre would be more common in mild cognitive impairment with Lewy bodies (MCI‐LB) than MCI due to Alzheimer's disease (MCI‐AD).

**Methods:**

MCI patients (*n* = 89) underwent longitudinal clinical assessment with differential classification of probable MCI‐LB, possible MCI‐LB, or MCI‐AD, with objective autonomic function testing at baseline. Blood pressure and heart rate responses to active stand and Valsalva manoeuvre were calculated from beat‐to‐beat cardiovascular data, with abnormalities defined by current criteria, and age‐adjusted group differences estimated with logistic models.

**Results:**

Orthostatic hypotension and abnormal heart rate response to orthostatic challenge were not more common in probable MCI‐LB than MCI‐AD. Heart rate abnormalities were likewise not more common in response to Valsalva manoeuvre in probable MCI‐LB. An abnormal blood pressure response to Valsalva (delayed return to baseline/absence of overshoot after release of strain) was more common in probable MCI‐LB than MCI‐AD. In secondary analyses, magnitude of blood pressure drop after active stand and 10‐s after release of Valsalva strain were weakly correlated with cardiac sympathetic denervation.

**Conclusions:**

Probable MCI‐LB may feature abnormal blood pressure response to Valsalva, but orthostatic hypotension is not a clear distinguishing feature from MCI‐AD.

## Key points

- There is limited evidence of early autonomic function abnormalities in prodromal dementia with Lewy bodies.
- This may suggest that there is a variable staging of symptom onset in early Lewy body disease, rather than the deterministic ‘body first’ staging previously theorised.

## 1 INTRODUCTION

Autonomic symptoms are common features of the synucleinopathies, including Parkinson's disease and dementia with Lewy bodies (DLB), and may include changes in heart rate (HR), blood pressure, temperature regulation, skin response, urination and digestion.^1^ Orthostatic hypotension (OH) in particular may be more common in DLB compared to other synucleinopathies.^2^

OH is characterised by a significant drop in blood pressure (BP) when moving from the supine position to standing upright.^3^ A reduction in orthostatic blood pressure without a compensatory increase in heart rate indicates a neurogenic cause of the OH.^4^ OH may cause symptoms such as light‐headedness, loss of balance, fatigue, or nausea, though a symptomatic response to OH is not required for diagnosis.^5^ Aside from orthostatic challenge, abnormal BP and HR responses may also be elicited by the Valsalva manoeuvre (VM); as with the orthostatic challenge, abnormal responses may present as an exaggerated change in BP or HR in response to VM, or a slowed return to baseline/lack of overshoot after release of strain.^6^

OH and its related symptoms may be of relevance to the screening and diagnosis of DLB, and contribute to the worse typical prognosis of this compared to Alzheimer's disease (AD), being linked to shorter survival time^7^; shorter survival being a common feature of DLB.^8^ However, OH is also reasonably common in AD and the normal ageing population, and so is not specific to DLB^9^: AD, which often co‐occurs in DLB, is itself associated with pathological changes to autonomic nuclei of the reticular formation,^10^ and so may also contribute to autonomic symptoms through a mixed pathology.

Autonomic symptoms in DLB such as OH may reflect the widespread cholinergic dysfunction common of Lewy body disease,^11^ or the presence of Lewy pathology (Lewy bodies and Lewy neurites) in the peripheral nervous system. Degeneration of the sympathetic nervous system is an early feature of DLB demonstrated by abnormalities in cardiac 123I‐metaiodobenzylguanidine (MIBG) scintigraphy,^12^ a finding also observed in the cognitive prodrome of DLB: mild cognitive impairment (MCI) with Lewy bodies (MCI‐LB).^13^

In both the dementia and prodromal stages of DLB, sympathetic denervation may be quantified by reduced uptake of MIBG to the heart relative to the mediastinum (heart:mediastinum ratio; HMR). As a reduced HMR has been demonstrated to often be present in the MCI stage of disease,^13^ MCI‐LB may also feature early autonomic dysfunction such as OH. However, this has not been examined in a prospective sample of MCI‐LB with objective measurement of OH, or cardiovascular response to VM.

We therefore aimed to investigate whether objectively measured cardiovascular dysfunctions would be an early feature of MCI‐LB, which might help distinguish this from normal ageing and MCI due to AD (MCI‐AD). We hypothesised that probable MCI‐LB would be more likely than MCI‐AD to feature abnormal blood pressure and heart rate responses to orthostatic challenge and Valsalva manoeuvre.

## 2 METHODS

### 2.1 Participants

As previously described,^14^ patients were recruited from older persons' health, psychiatry and neurology services in North East England with a health service diagnosis of MCI, and reported presence of either any core clinical features of LB disease (parkinsonism, REM sleep behaviour disorder, complex visual hallucinations, and fluctuating attention and cognition), or any other supportive features sensitive to LB disease but also found in AD (e.g. changes in mood, anxiety, autonomic symptoms, or sleep disturbance). All were aged ≥60 years and required to be medically stable. All provided written informed consent to undergo further screening, and were excluded from further assessment if they had dementia, no objective cognitive impairment, or evidence of any frontotemporal or vascular aetiology.

### 2.2 Assessment and imaging

Participants underwent repeated cognitive and clinical assessment at 1 year after baseline, with adaptive scheduling of assessments every 12–18 months thereafter due to the COVID‐19 pandemic. These assessments provided detailed notes on cognitive and clinical function, and any other clinical symptoms, to be reviewed by a three‐person expert panel of old age psychiatrists (AJT, PCD, JPT) for diagnosis (see below). Assessments included a number of detailed cognitive and clinical measures as previously reported, with relevant measures to this analysis being the Addenbrooke's Cognitive Examination – Revised (ACE‐R) and the Unified Parkinson's Disease Rating Scale – Motor Subscale (UPDRS) to assess global cognitive impairment and motor impairment, respectively.

At baseline, and at first‐year follow‐up, all participants were offered 123I–2β‐carbomethoxy‐3β‐(4‐iodophenyl)‐*N*‐(3‐fluoropropyl) nortropane single‐photon emission computed tomography (FP‐CIT) imaging. Images were rated as normal or abnormal (unilateral or bilateral reduction in tracer uptake to the striatum) by an experienced panel of image analysts blind to any clinical information, as previously described.^15^ Ratings were updated if there was any change at follow‐up.

All participants were similarly offered MIBG cardiac scintigraphy at baseline, as previously described.^13^ HMR was quantified from delayed images, and classified as abnormal given a HMR <1.85, with this cut‐off value derived from locally recruited cognitively healthy comparators.^16^

Of this sample, three did not complete MIBG imaging (declined or were ineligible), and two declined FP‐CIT imaging. Imaging results were then incorporated into baseline diagnosis and annual diagnostic reviews.

Structural and functional MRI were conducted at baseline, as previously reported.^17^, ^18^ Any significant cerebrovascular disease on MRI was cause for exclusion as a possible vascular cognitive impairment.

### 2.3 Cognitive impairment diagnosis and differential classification

As previously described,^14^ at baseline and after each follow‐up the consensus panel rated the presence of either MCI,^19^ given presence of cognitive impairment but maintained independent function, or all‐cause dementia^20^ when there was evidence of a loss of independent function related to cognitive impairment.

The panel also rated the presence or absence of each of four core clinical features of DLB: parkinsonism, REM sleep behaviour disorder, complex visual hallucinations, and cognitive fluctuations. Incorporating FP‐CIT and MIBG imaging results, MCI cases were then differentially classified as either MCI‐AD (MCI with no core clinical features of DLB, normal MIBG and FP‐CIT imaging, and evidence of decline that was characteristic of Alzheimer's disease, i.e., they met the additional NIA‐AA criterion of ‘etiology of MCI consistent with Alzheimer's disease pathophysiologic process’^19^), possible MCI‐LB (MCI with one core clinical feature of DLB and normal imaging, or MCI with no core clinical features of DLB and abnormal imaging), or probable MCI‐LB (MCI with two or more core clinical features of DLB, or one core clinical feature with abnormal imaging) according to current research criteria.^21^ These diagnoses were updated after follow‐up as and when new clinical information arose. When dementia was observed, a diagnosis of AD or DLB was made by the same panel consistent with current consensus criteria.^20^, ^22^

### 2.4 Procedure

At baseline, all patients were offered autonomic function testing, measured using the CNSystems Task Force ® Monitor. This was administered by a medically qualified researcher (RD, SL) in a single session consisting of a 10‐min supine rest to obtain baseline measures, followed by 3‐min of active standing. Participants rested quietly for 1–7 min as needed (median = 2 min) to enable cardiovascular measures to stabilise before performing 2–3 VM. Participants blew into a closed circuit, connected to a manometer, for 15 s, aiming for an exhalation pressure of 30–40 mmHg. In cases where there was interruption to either the resting or testing procedure, this was recorded by the clinical researcher and the procedure repeated as appropriate. HR was measured concurrently by electrocardiogram using standard limb leads I or II, sampling at 1 kHz.

### 2.5 Data cleaning

Cardiovascular data was exported into individual case files. Signal artefact and ectopic beat data were identified by comparing systolic and diastolic BP, and R‐R interval (RRI) measurements at each beat to those of the preceding beat; if the measurement differed from the immediately preceding beat by 10% or greater (e.g. a beat‐to‐beat increase in HR from 60 to >66 BPM), they were flagged as potential artefacts. Where a lagged beat could not be obtained, such as if the preceding beat measurement was missing, then the lagged value was estimated by spline interpolation. Any interpolated values were not included in subsequent analysis. Possible artefacts were manually inspected and removed, and the resulting patient‐level data visually inspected for validity. Participants with persistent artefacts such that a stable baseline value could not be identified, or individual recording blocks with over 50% of data missing, were deemed to be unusable and excluded from further analysis.

### 2.6 Orthostasis

Continuous BP data was converted into 5‐s means. The orthostatic BP drop was calculated by subtracting the nadir 5‐s mean standing systolic and diastolic BP from the respective mean BP of the final 5‐s of supine rest. An abnormal BP response to standing was considered if the orthostatic BP decreased by ≥20 mmHg systolic BP or by ≥10 mmHg in diastolic BP. If the participant had supine hypertension (≥160 mmHg at rest) then a drop in systolic BP of ≥30 mmHg was considered abnormal.^3^ Initial OH (within 15 s of active standing) was not considered abnormal due to uncertainty of its clinical significance.^23^

In those who met the BP criteria for OH, the ratio of the change in heart rate and systolic BP (SBP) from baseline to 3‐min was calculated (ΔHR/ΔSBP). The mean HR of the final 5‐s of supine rest was considered the baseline HR. The mean HR for the final 5‐s of standing was calculated. A ΔHR/ΔSBP ratio of <0.492 bpm/mmHg has a high sensitivity and specificity for distinguishing neurogenic OH (nOH) and non‐neurogenic OH.^6^ HR changes were not calculated for individuals taking beta‐ or alpha‐blockers, non‐dihydropyridine calcium channel blockers, central alpha‐2 agonists (clonidine) or antiarrhythmic agents. HR changes were also not calculated for those with arrhythmias or pacemakers.

The orthostatic RRI 30:15 ratio, from Ewing and Clark's autonomic battery^24^ was calculated for all participants. Using the raw RRI data, the longest RRI at 30‐s of standing and the shortest RRI at 15‐s of standing was calculated. The following age‐adjusted normal values^24^, ^25^ were used:

- 61–65 years ≥ 1.02,
- 66–70 years ≥ 1.01,
- ≥71 years ≥ 1.00.

### 2.7 Valsalva

To select which Valsalva manoeuvre (VM) to use for analysis, the data was visually inspected and the VM which achieved the greatest degree of hypotension in phase 2 of the manoeuvre was selected. Participants who were unable to successfully perform the VM were excluded from this analysis, but retained for active stand analyses. The ratio of the longest RRI in phase 4 (within 10 s of release of strain) to the shortest RRI during phase 2 or 3 was calculated. The ratio was considered normal^24^, ^25^ if:

- 61–65 years ≥ 1.08,
- 66–70 years ≥ 1.04,
- ≥71 years ≥ 1.00.

The BP response to VM was considered normal if the systolic BP had exceeded the baseline BP (before phase 1 of VM) within 10‐s of release of strain.

### 2.8 Statistical analysis

Binary logistic models estimated differences between MCI‐AD and probable MCI‐LB in presence of each of four outcomes: orthostatic hypotension, abnormal HR response to orthostasis, abnormal BP response to VM, and abnormal HR response to VM. These were adjusted for age (mean centred), and the use of cholinesterase inhibitors, since these may be independently associated with OH.^26^ The possible MCI‐LB were included for additional context, but were not interpreted in relation to the primary hypothesis. There was no statistical adjustment for multiple comparisons.

Secondary analyses were undertaken with linear models to assess any associations between the magnitude of any BP abnormalities, and either MCI subtype or degree of MIBG abnormality.

## 3 RESULTS

Of 103 participants, 89 provided useable cardiovascular data for at least one of the assessments. Baseline demographic and autonomic characteristics are described in Table 1; data completeness for the orthostatic challenge and Valsalva procedures are also included, as are numbers of exclusions from heart rate analyses due to medication or arrhythmia. None of the 89 retained patients had a pacemaker, or were taking central alpha‐2 agonists, antiarrhythmic agents, midodrine or fludrocortisone.

**TABLE 1:** Characteristics of sample at baseline

Characteristics	MCI‐AD, *N* = 35^a^	Poss. MCI‐LB, *N* = 17^a^	Prob. MCI‐LB, *N* = 37^a^
Age	76 (8)	75 (8)	75 (6)
Female sex	20 (57%)	8 (47%)	5 (14%)
ACE‐R score	83 (8)	79 (11)	84 (9)
Resting systolic (mmHg)	110 (23)	114 (18)	118 (20)
Resting diastolic (mmHg)	64 (16)	64 (14)	69 (12)
Resting heart rate (bpm)	66 (12)	64 (12)	64 (19)
Using cholinesterase inhibitors	5 (15%)	3 (19%)	16 (44%)
Using levodopa	0 (0%)	0 (0%)	3 (8.1%)
Reasons for exclusion from heart rate analyses			
Receiving alpha blockers	0 (0%)	0 (0%)	1 (2.7%)
Receiving beta blockers	6 (18%)	5 (29%)	6 (16%)
Receiving calcium channel blockers	5 (15%)	5 (29%)	11 (30%)
Any arrhythmia	2 (5.9%)	1 (5.9%)	2 (5.4%)
Missing data			
Unusable orthostatic hypotension data	2 (5.7%)	0 (0%)	1 (2.7%)
Unusable orthostatic challenge heart rate data	5 (14%)	1 (5.9%)	2 (5.4%)
Unusable valsalva manoeuvre blood pressure data	7 (20%)	1 (5.9%)	5 (14%)
Unusable valsalva manoeuvre heart rate data	8 (23%)	1 (5.9%)	5 (14%)

^a^ Mean (SD); *n* (%).

The observed rates of BP and HR abnormalities in response to the orthostatic challenge and VM are reported in Table 2. As there is overlap between different HR exclusion criteria, or between medical exclusions and missing data, their sum is greater than the total sum of HR exclusions or data missingness (e.g. some participants may receive both beta blockers and calcium channel blockers, or may receive beta blockers and also provide unusable HR data).

**TABLE 2:** Occurrence of abnormal cardiovascular responses to orthostatic challenge and Valsalva manoeuvre

Orthostatic challenge	MCI‐AD	Poss. MCI‐LB	Prob. MCI‐LB
Abnormal BP response (OH)	6/33 (18%)	8/17 (47%)	7/36 (19%)
Abnormal RRI30:15	6/26 (26%)	2/7 (29%)	3/19 (16%)
Neurogenic OH	5/32 (16%)	6/16 (38%)	5/35 (15%)
Valsalva manoeuvre			
Abnormal systolic BP response	3/28 (11%)	5/16 (31%)	14/32 (44%)
Abnormal heart rate response	3/21 (14%)	1/7 (14%)	4/19 (21%)

Abbreviations: BP, blood pressure; OH, orthostatic hypotension.

There were no significant differences between those with or without abnormal BP and HR responses to orthostasis or VM in age‐adjusted cognitive function (measured with the Addenbrooke's Cognitive Examination – Revised) or motor function (measured with the Unified Parkinson's Disease Rating Scale – Motor Subscale).

Results of the logistic models are presented in Table 3; these suggested that there was no clear evidence that abnormal BP and HR response to orthostasis were significantly more or less likely in probable MCI‐LB than MCI‐AD, nor was an abnormal HR response to VM. The fourth model indicated that odds of abnormal BP responses to VM (a drop in SBP which did not return to baseline within 10 s of release of strain) were approximately six times higher in probable MCI‐LB than MCI‐AD, while adjusting for use of cholinesterase inhibitors. Exploratory analyses of the smaller possible MCI‐LB group identified a significant over‐representation of OH in this group.

**TABLE 3:** Binary logistic models for presence of each autonomic outcome

	Orthostatic challenge			Valsalva manoeuvre	
	Orthostatic hypotension	Abnormal heart rate	Neurogenic OH	Abnormal blood pressure	Abnormal heart rate
Intercept: MCI‐AD^a^	0.19 (0.06–0.46)	0.44 (0.15–1.15)	0.18 (0.06–0.44)	0.10 (0.02–0.33)	0.14 (0.03–0.47)
Probable MCI‐LB versus MCI‐AD^b^	0.95 (0.25–3.59)	0.59 (0.10–2.97)	0.75 (0.17–3.21)	6.40 (1.63–33.22)	0.93 (0.12–6.79)
Possible MCI‐LB versus MCI‐AD^b^	4.81 (1.24–20.62)	0.95 (0.11–6.05)	3.49 (0.83–15.64)	4.20 (0.82–25.33)	0.96 (0.04–9.81)
Age (per year)^b^	1.07 (0.99–1.16)	0.95 (0.85–1.05)	1.04 (0.96–1.13)	1.08 (1.00–1.18)	0.95 (0.82–1.08)
Cholinesterase inhibitor use^b^	2.04 (0.59–7.20)	0.44 (0.05–2.40)	1.80 (0.45–7.05)	1.60 (0.48–5.31)	1.66 (0.24–11.45)
Observations	82	47	47	72	45

*Note*: Estimates represent adjusted baseline odds or odds ratios of each autonomic symptom presence, with 95% confidence intervals.

Abbreviation: OH, orthostatic hypotension.

^a^ Baseline Odds (95% CI).

^b^ Odds Ratio (95% CI).

Due to the gender imbalance across diagnostic groups, we conducted an additional sensitivity analysis to assess whether there was any gender‐associated effect. This did not meaningfully change any of the observed findings, and there was no main effect of gender itself on any autonomic outcome.

A secondary analysis explored the associations between MIBG HMR, a measure of sympathetic denervation, and cardiovascular responses to orthostasis and VM.

**FIGURE 1:**
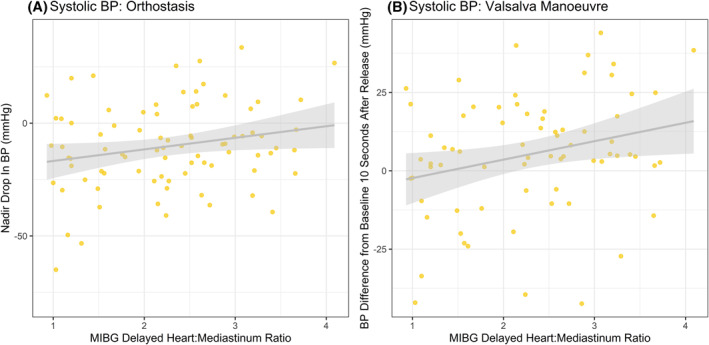
Associations between metaiodobenzylguanidine heart:mediastinum ratio in overall mild cognitive impairment group and (A) Orthostatic drop in systolic blood pressure after active stand (B) difference from baseline systolic blood pressure 10 s after release of Valsalva strain (negative value corresponds to failure to return to baseline, positive value corresponds to overshoot of baseline)

There was a marginally significant association (see Figure 1) between higher HMR and an attenuated SBP drop at standing (Standardised Beta = 0.22, 95% CI = 0.0–0.44, *p* = 0.047; corresponding to 5.13 mmHg higher nadir SBP per one‐unit higher HMR, 95% = 0.06–10.19 mmHg), and for VM, a significant association between higher HMR and SBP recovery to baseline or overshoot within 10‐s of release of strain (Standardised Beta = 0.26, 95% CI = 0.03–0.49, *p* = 0.027; 5.88 mmHg higher SBP per one‐unit increase in HMR, 95% CI = 0.68–11.09 mmHg) within MCI overall.

This pattern was partially replicated in the probable MCI‐LB sub‐group specifically, with significantly attenuated orthostatic drop with higher HMR (Standardised Beta = 0.36, 95% CI = 0.02–0.69, *p* = 0.038), but no significant association between HMR and recovery to baseline BP after release of VM strain (Standardised Beta = 0.07, 95% CI = −0.32–0.45, *p* = 0.727).

Finally, considering that the groups might differ not only in the presence/absence of BP abnormalities, but in the magnitude of BP drop, we conducted an exploratory analysis of the differences between MCI sub‐groups in the magnitude of SBP drop in response to active stand, and difference in SBP from baseline 10 s after release of VM strain, controlling for age, cholinesterase inhibitor use, and baseline SBP. Probable MCI‐LB did not significantly differ from MCI‐AD in the magnitude of SBP drop after active stand (Estimate = −5.5, 95% CI = −16.35–5.32), but had a significantly lower SBP (Estimate = −14.39, 95% CI = −24.60 to −4.19) 10 s after release of VM strain than did MCI‐AD, who generally displayed some degree of SBP overshoot (Estimated marginal mean for MCI‐AD = 11.8 mmHg higher than pre‐VM baseline, 95% CI = 3.9–19.6 mmHg).

## 4 DISCUSSION

We aimed to assess whether probable MCI‐LB would have a greater probability of presenting with early abnormalities in autonomic response to orthostasis and Valsalva manoeuvre than MCI‐AD, as is seen in DLB compared with AD. An abnormal BP response to Valsalva was significantly more common in probable MCI‐LB than in MCI‐AD. However contrary to our hypothesis, OH and neurogenic OH specifically were not more common in MCI‐LB than MCI‐AD, nor were abnormal HR responses to orthostatic challenge or Valsalva manoeuvre.

Inconsistencies between the absence of greater OH in MCI‐LB, but presence of abnormal SBP response to VM, suggests that, though statistically significant, the latter finding requires validation if there is to be more confidence in its veracity; as demonstrated by the wide confidence intervals, there is considerable uncertainty as to the size of the effect. OH was more common in possible MCI‐LB than MCI‐AD; however, as a small group with uncertain diagnosis this does not clearly support our hypothesis. As neurogenic OH was not more common in possible MCI‐LB, we do not consider this to be a robust finding. MIBG HMR was weakly associated with the degree of SBP dysfunction across the whole sample, supporting that BP abnormalities in MCI may be features of sympathetic denervation, however this was evidently not sufficient to translate into a greater risk of OH in MCI‐LB.

As the MCI‐AD group were recruited to the study due to the presence of supportive clinical features of MCI‐LB, they may have a more LB‐like clinical profile than typical of MCI‐AD more broadly. However, the overall rates of OH in this sample, around 20%, do not differ considerably from those in the general older population.^27^ It remains possible however that some cases of MCI‐AD may in fact represent clinically undetected MCI‐LB, given the absence of AD biomarkers available. It may be that at this early stage any differences in cardiovascular responses are too subtle to manifest in different rates of clinical OH across MCI subtypes; alternatively, this may reflect the generally better health of clinical research participants than that of the wider clinical population they represent, as poor health is itself a barrier to research participation.

MCI‐LB patients were more likely to be in receipt of calcium channel blockers and have previously been shown to be more likely to be in receipt of cholinesterase inhibitors^14^; the administration of cholinesterase inhibitors in MCI‐LB is consistent with local recommendations for managing other clinical features of LB disease,^28^ even in the absence of dementia. While efforts were made to adjust for this, as with any observational study, differences in medication use might contribute to obscuring true group differences through either therapeutic effects or exclusion of participants with specific characteristics.

These data may also be limited more generally by the methods of collection. While the blood pressure measurements calibrate finger cuff BP against arm cuff oscillatory BP, neither reflects the gold standard: the Task Force ® Monitor has an accuracy of +/− 5 mmHg in the range of interest (50–250 mmHg). Artefacts were common and many participants were unable to provide data either through excessive artefacts or signal loss, or due to their medical history: such missing data may well reflect processes of interest (e.g. invalid BP measures due to dysautonomia related to neurodegeneration). Correct performance of the Valsalva manoeuvre is dependent on participant technique, and cognitive impairment could impose an additional barrier to this.

## 5 CONCLUSIONS

Probable MCI‐LB may feature a delayed return to baseline BP after Valsalva, but there was no clear evidence that MCI‐LB are more likely to feature OH, neurogenic OH, or heart rate abnormalities after active stand or Valsalva.

## CONFLICT OF INTEREST

The authors have no conflict of interest to declare.

## Data Availability

Data supporting this analysis are available from the corresponding author Calum A. Hamilton.
